# ERX-208 Induces Endoplasmic Reticulum Stress and Suppresses DNA Damage Repair to Enhance Chemotherapy Sensitivity

**DOI:** 10.1158/2767-9764.CRC-26-0421

**Published:** 2026-10-29

**Authors:** Durga Meenakshi Panneerdoss, Khaled Mohamed Nassar, Baskaran Subramani, William Cole Arnold, Sai Pranathi Vedagiri Venkata, Joe Hernandez, Behnam Ebrahimi, Sonal R. Chaudhari, Gaurav Sharma, Tae-Kyung Lee, Scott Elmore, Henry Neal, Uday P. Pratap, Gangadhara R. Sareddy, Edward R. Kost, Jung-Mo Ahn, Ganesh V. Raj, Ratna K. Vadlamudi

**Affiliations:** 1 Department of Obstetrics and Gynecology, University of Texas Health San Antonio, San Antonio, Texas.; 2 Department of Chemistry and Biochemistry, University of Texas at Dallas, Richardson, Texas.; 3 Mays Cancer Center, San Antonio, Texas.; 4 EtiraRx, Dallas, Texas.; 5 Audie L. Murphy South Texas Veterans Health Care System, San Antonio, Texas.

## Abstract

Ovarian cancer remains the most lethal gynecologic malignancy, driven in part by intrinsic and acquired therapeutic resistance to DNA-damaging chemotherapies. We previously demonstrated that ERX-208 targets the protein encoded by lysosomal acid lipase to induce catastrophic endoplasmic reticulum (ER) stress in ovarian cancer models. As ERX-class compounds advance to clinical development, rational combination strategies are needed to maximize therapeutic efficacy. To address this question, we conducted an unbiased *in vitro* screen of the combination of ERX-208 with 147 FDA-approved agents. We identified that DNA-damaging chemotherapies, including anthracyclines, oxaliplatin, and paclitaxel, were most synergistic in combination with ERX-208. Reanalysis of our transcriptomic data in ovarian cancer cells revealed coordinated suppression of DNA damage repair, DNA replication, and checkpoint pathways following ERX-208 treatment. Together, these data support a narrative that ERX-208 may selectively suppress DNA repair pathways and potentiate cytotoxicity by DNA-damaging agents. To test this hypothesis, we used platinum and paclitaxel, two standard-of-care agents in ovarian cancer. We found that ERX-208 synergized with platinum and paclitaxel across multiple ovarian cancer models, enhancing apoptosis and suppressing proliferation, clonogenicity, and invasion. Mechanistically, combination treatment increased DNA damage and amplified ER stress and apoptotic signaling. Importantly, ERX-208–based combinations showed enhanced antitumor activity in patient-derived explant, and xenograft models. These findings support a model in which ERX-208 impairs DNA damage response pathways and sensitizes ovarian cancer cells to DNA-damaging chemotherapy. Collectively, these findings provide a strong rationale for the combination strategy in clinical translation.

**Significance:**

This study demonstrates the clinical utility of ERX-208 in enhancing chemotherapy efficacy in ovarian cancer. By inducing sustained ER stress and a functional DNA repair–deficient state, ERX-208 sensitizes treatment-naïve and treatment-resistant tumors to platinum and taxanes. Findings support the rapid clinical development of ERX-class combinations, offering a rationale for pairing with standard regimens. This strategy may enable dose reduction, overcome resistance, expand options, and support biomarker-guided patient selection.

## Introduction

Epithelial ovarian cancer remains the most lethal gynecologic malignancy in the United States. Although most patients initially respond to cytoreductive surgery followed by platinum- and taxane-based chemotherapy, more than 90% ultimately develop recurrent, drug-resistant disease, resulting in a poor long-term prognosis and 5-year survival rates below 20% (1). Extensive intra- and intertumoral heterogeneity and high genomic instability contribute to therapy resistance and disease recurrence (2). Platinum-resistant ovarian cancer remains largely incurable, highlighting the urgent need for new strategies to enhance the efficacy of existing DNA-damaging chemotherapeutics (3, 4).

Ovarian cancer cells exhibit high rates of cellular proliferation, leading to sustained demands for new protein synthesis and an elevated basal level of endoplasmic reticulum (ER) stress (5). Somatic mutations and genomic instability in advanced ovarian cancer lead to an accumulation of misfolded proteins, compensatory activation of the unfolded protein response (UPR; ref. 6), and further enhancement of ER stress in ovarian cancer cells. Consequently, elevated expression of key UPR regulators, including GRP78, eIF2α, PERK, and ATF6, is seen in advanced ovarian cancer and correlates with both disease progression and poor patient outcomes (7). Genome-wide functional screens have identified individual components of the ER stress pathway as critical vulnerabilities in ovarian cancer (8–10). Together, these emerging findings suggest that the elevated basal ER stress in ovarian cancer represents a therapeutically exploitable vulnerability.

We had previously shown that a small molecule, ERX-41, induces ER stress and apoptosis in multiple cancer cell types (including ovarian) while exhibiting no evidence of cytotoxicity in normal cells (11). Unbiased CRISPR screens identified lysosomal acid lipase A (LIPA) as the critical target of ERX-41. We subsequently developed a potent tris-benzamide analog of ERX-41, ERX-208, which had robust LIPA-dependent activity against multiple molecular subtypes of ovarian cancer (12). ERX-208 suppresses tumor growth in patient-derived explant (PDE) and patient-derived xenograft (PDX) models, establishing LIPA targeting as a viable therapeutic strategy. Although prior studies established ERX-208 as a potent LIPA-targeting ER stress inducer with single-agent activity in ovarian cancer, the optimal clinical deployment of ERX-208 remains unknown. As ERX-class compounds advance toward clinical development, the identification of rational combination partners becomes increasingly important.

In this study, we performed an unbiased screen of 147 FDA-approved agents to identify therapies that synergize with ERX-208 in ovarian cancer. We show that DNA-damaging chemotherapies (13–15) emerge as the dominant class of synergistic agents. Our mechanistic studies identified the ability of ERX-208 to functionally impair DNA repair capacity and reprogram stress–response pathways, as well as its ability to potentiate chemotherapy-induced cell death in ovarian cancer. Taken together, these data provide mechanistic and translational evidence that ERX-208 enhances chemotherapy sensitivity across multiple clinically relevant ovarian cancer models.

## Materials and Methods

### Cell lines and reagents

Established ovarian cancer cell lines ES2 (RRID: CVCL_3509), SKOV3 (RRID: CVCL_0532), A2780 (RRID: CVCL_0134), TOV21G (RRID: CVCL_3613), and TOV112D (RRID: CVCL_3612) were obtained from the American Type Culture Collection (ATCC) and were maintained using ATCC-recommended media. OVCAR8 (RRID: CVCL_1629) cells were obtained from the National Cancer Institute (NCI) Division of Cancer Treatment and Diagnosis (DCTD) repository. The generation and characterization of SKOV3-LIPA knockout (KO) cells have been described previously (12). All cancer cell lines were authenticated by short tandem repeat profiling and screened for *Mycoplasma* contamination using a PCR-based assay. Cell lines were used within 10 to 15 passages. Ovarian cancer patient-derived primary cells and ascites-derived cells were obtained from the Ob/Gyn Tissue Core and have been described previously (16). The antibodies for phospho-eIF2α (#3398; RRID: AB_2096481), RAD51 (#8875; RRID: AB_2721109), phospho–histone H2AX (Ser139; #9718; RRID: AB_2118009), GRP78 (#3177; RRID: AB_2119845), and vinculin (#13901; RRID: AB_2728768) were purchased from Cell Signaling Technology. The Ki-67 antibody (ab16667; RRID: AB_302459) was acquired from Abcam. The LAL (LIPA) antibody (sc-58374; RRID: AB_784329) was obtained from Santa Cruz Biotechnology. The β-actin antibody (#A3854; RRID: AB_262011) was purchased from Sigma-Aldrich.

### Cell viability assays (CellTiter-Glo and MTT)

Cell viability was measured using either the CellTiter-Glo Luminescent Cell Viability Assay (Promega) or the 3-(4,5-dimethylthiazol-2-yl)-2,5-diphenyltetrazolium bromide (MTT) colorimetric assay according to established protocols (12). Cells were seeded in 96-well plates (2 × 10^3^ cells/well) and treated with ERX-208, cisplatin, carboplatin, paclitaxel, or combinations for 72 hours. For CellTiter-Glo, plates were equilibrated to room temperature, and CellTiter-Glo reagent was added at a ratio of 1:1 to each well, mixed, and incubated for 10 minutes before luminescence measurement. For MTT assays, MTT solution (5 mg/mL) was added for 3 hours, followed by solubilization in DMSO and absorbance measurement at 570 nm.

### Approved oncology drug set IX screening

**Figure 1.**
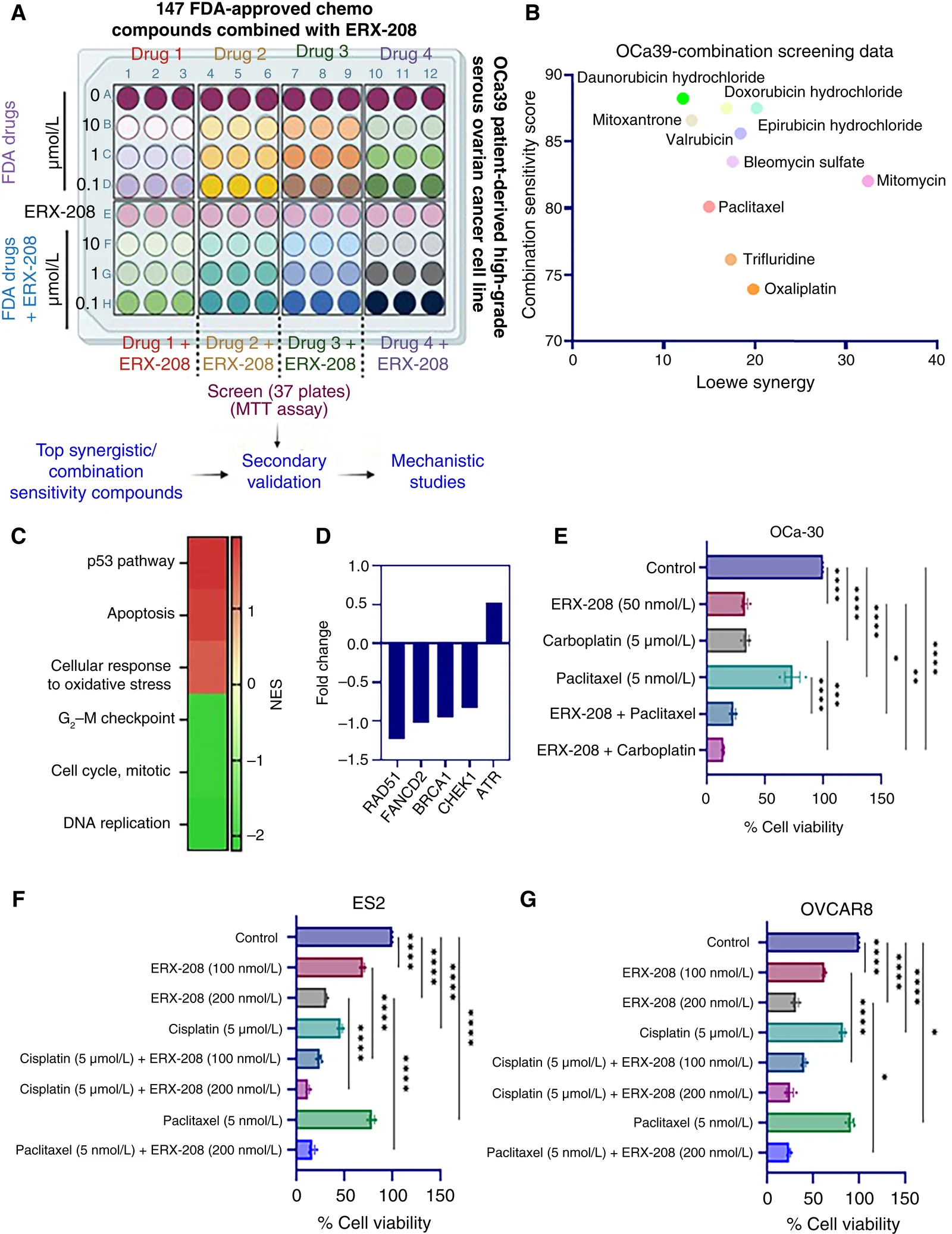
ERX-208 sensitizes ovarian cancer cells to DNA-damaging agents. **A,** Schematic overview of the unbiased drug combination screening strategy. ERX-208 was combined with a library of 147 FDA-approved chemotherapeutic agents and screened using an MTT viability assay in OCa-39 cells. **B,** Quantitative assessment of ERX-208 drug interactions. The scatter plot shows combination sensitivity and synergy metrics calculated using SynergyFinder Plus with Loewe additivity modeling, identifying compounds that exhibit enhanced efficacy in combination with ERX-208. **C,** Gene set enrichment analysis of DDR, replication, and checkpoint pathways following ERX-208 treatment. The bubble plot shows normalized enrichment scores (NES) for significantly enriched pathways, with negatively enriched pathways (green) indicating repression of DNA repair, replication, and cell-cycle programs and positively enriched pathways (red) indicating activation of stress and apoptotic responses. **D,** Differential regulation of DNA repair pathway genes in SKOV3 cells following ERX-208 treatment. **E,** Viability of OCa-30 patient-derived ovarian cancer cells treated with ERX-208 (50 nmol/L), carboplatin (5 μmol/L), paclitaxel (5 nmol/L), or combination treatments. Combined ERX-208 + carboplatin or ERX-208 + paclitaxel produced greater cytotoxicity than monotherapies. **F** and **G,** Cell viability analysis of ES2 (**F**) and OVCAR8 (**G**) ovarian cancer cells treated with ERX-208 (100 or 200 nmol/L), cisplatin (5 μmol/L), paclitaxel (5 nmol/L), or their combinations for 72 hours. Combination treatment significantly reduced viability compared with single-agent treatments. Data show mean ± SEM, with statistical significance indicated as *, *P* < 0.05; **, *P* < 0.01; ***, *P* < 0.001; ****, *P* < 0.0001.

A panel of 147 FDA-approved anticancer drugs (https://dctd.cancer.gov/drug-discovery-development/reagents-materials/vialed-plated-compounds-0) was obtained from the NCI at a stock concentration of 10 mmol/L. The primary drug screen was performed in patient-derived primary ovarian cancer OCa-39 cells using an MTT cell viability assay. OCa-39 cells were seeded into 96-well plates at a density of 2 × 10^3^ per well and allowed to adhere overnight. Each compound was tested both alone and in combination with ERX-208 to assess potential synergistic effects. ERX-208 was used at a constant concentration of 100 nmol/L and combined with each approved compound at final concentrations of 0.1, 1, and 10 μmol/L for 72 hours. Following treatment, cell viability was assessed using the MTT assay according to the previously described protocol (12). The screening workflow was designed to identify top synergistic or sensitizing compounds for subsequent combination testing with ERX-208, followed by secondary validation and mechanistic studies, as illustrated in the screening schema (Fig. 1A).

### Colony formation assays

A total of 500 to 1,000 cells were plated in six-well plates and treated with ERX-208 alone or in combination with chemotherapeutic agents for 10 to 14 days as described (12). Colonies were washed with phosphate-buffered saline (PBS), fixed in methanol for 20 minutes, and stained with 0.5% crystal violet. Plates were rinsed, and colonies containing >50 cells were counted manually or using ImageJ.

### Caspase-Glo 3/7 assay

Apoptotic activity was quantified using the Caspase-Glo 3/7 Assay (Promega). Cells were seeded in white 96-well plates and treated with indicated agents for 24 hours. Caspase-Glo reagent was added at a ratio of 1:1 to each well and incubated for 30 minutes at room temperature. Luminescence was recorded using a Promega GloMax microplate reader, and caspase activity was normalized to vehicle controls.

### Invasion assays

Cell invasion was assessed using Matrigel-coated Transwell chambers (Corning). A total of 5 × 10^4^ cells in serum-free medium were seeded into the upper chamber with ERX-208 and/or chemotherapeutic agents. The lower chambers contained medium with 10% FBS as the chemoattractant. After 22 hours, noninvading cells were removed, and invading cells were fixed, stained with crystal violet, and imaged. Invaded cells were quantified using ImageJ.

### Western blot analysis

Total cell lysates were prepared using RIPA buffer containing protease and phosphatase inhibitors. Protein concentrations were determined using the Bio-Rad DC Protein Assay. Equal amounts of protein (20–40 μg) were resolved on SDS-PAGE gels and transferred to either polyvinylidene difluoride or nitrocellulose membranes. Membranes were blocked in 5% nonfat milk and incubated with primary antibodies against RAD51, phospho-H2AX (γH2AX), LIPA, and vinculin/β-actin (loading controls). Horseradish peroxidase (HRP)–conjugated secondary antibodies and enhanced chemiluminescence were used for detection.

### RT-PCR for XBP1 splicing

Total RNA was isolated using TRIzol reagent. cDNA synthesis was performed using reverse transcriptase and oligo-dT primers as described (12). XBP1 splicing (sXBP1) was assessed using primers flanking the IRE1α splice site. PCR products were separated on a 2.5% agarose gel to distinguish unspliced and spliced XBP1 isoforms.

### qRT-PCR analysis of ER stress markers and RNA sequencing analyses

RNA was isolated using TRIzol and reverse-transcribed using standard protocols. qRT-PCR was performed using SYBR Green Master Mix on a real-time PCR system. Gene-specific primers targeting ATF3, ATF4, CHOP/DDIT3, PERK, XBP1, and HSPA5/BiP were used. Relative gene expression was calculated using the ΔΔCt method with GAPDH as the internal control. Previously generated RNA sequencing datasets from our group (12) were reanalyzed to identify pathways associated with chemotherapy sensitization.

### Immunofluorescence assay

Cells were seeded at a density of 2 × 10^4^ cells per well in eight-chamber glass slides and allowed to attach prior to treatment. Cells were then exposed to the indicated DNA-damaging agent for 24 hours. Following treatment, cells were washed with PBS and fixed with 4% paraformaldehyde for 15 minutes at room temperature. Fixed cells were washed 3 times with PBS for 5 minutes each and permeabilized with 0.25% Triton X-100 for 10 minutes. After permeabilization, cells were blocked with PBS containing 0.1% Tween 20 and 1% bovine serum albumin (BSA) for 30 minutes at room temperature. Cells were then incubated with primary antibodies against γH2AX and RAD51 (1:200 dilution) for 2 hours, followed by incubation with the appropriate fluorophore-conjugated anti-mouse and anti-rabbit secondary antibodies (1:200 dilution) for 1 hour in 1% BSA. After washing with PBS, nuclei were counterstained with DAPI and mounted using ProLong Gold Antifade Mountant (Molecular Probes, Life Technologies). Immunofluorescence images were acquired using a confocal microscope, and at least 100 cells were randomly scored per sample for the quantification of γH2AX and RAD51 foci.

### Alkaline comet assay (single-cell gel electrophoresis)

DNA damage was assessed by an alkaline comet assay using the published protocol (17). ES2 and OVCAR8 cells were treated for 20 hours with vehicle, ERX208 (200 nmol/L), cisplatin (5 μmol/L), paclitaxel (5 nmol/L), or the indicated combination treatments. Cells were trypsinized, resuspended as single-cell suspensions in PBS, and mixed with 1% low-melting-point agarose. The cell suspension was dropped onto microscope slides precoated with 1% normal-melting agarose and allowed to solidify at 4°C. Slides were incubated in freshly prepared alkaline lysis buffer (pH > 13) for 30 minutes at 4°C in the dark, and DNA was then unwound in alkaline electrophoresis buffer (pH 12.3) for 30 minutes. Electrophoresis was performed in the dark for 10 minutes at 4°C at 1 V/cm (∼40 mA). Slides were neutralized, dehydrated in 100% ethanol, air-dried, rehydrated, and stained with 1× SYBR Gold (Invitrogen #S11494). Comets were visualized by fluorescence microscopy, and DNA damage was quantified as the percentage of tail DNA (% tail DNA) using CometScore software.

### DNA damage response phosphorylation

DNA damage–associated protein phosphorylation was analyzed using the Human DNA Damage Response Phosphorylation Array C1 (cat. #AAH-PhoDDR-1-4). Nitrocellulose membranes prespotted with DNA damage response (DDR) protein antibodies were incubated with cell lysates. Bound proteins were detected using the provided biotinylated detection antibodies and chemiluminescence. Signal intensities were quantified using ImageJ software.

### PDE assays

Fresh deidentified ovarian tumor tissues were obtained under Institutional Review Board (IRB)–approved protocols from the University of Texas Health San Antonio (UTHSA) OBGYN tissue core (18). All samples were collected and distributed in accordance with IRB-approved protocols. The study was conducted in compliance with the Declaration of Helsinki and was approved by the IRB of UT Health San Antonio (IRB protocol HSC20190695N; approval date: September 3, 2019). Tumors were minced into 1 to 2 mm^3^ pieces and cultured on gelatin-sponge inserts in DMEM/F-12 supplemented with 10% FBS. Explants were treated with ERX-208, cisplatin, carboplatin, paclitaxel, or combinations for 72 hours. Tissues were fixed in 10% formalin and processed for paraffin embedding, followed by immunohistochemistry (IHC; ref. 12).

### Patient-derived organoid assays

Ovarian cancer patient-derived organoids (PDO) were established from deidentified fresh tumor samples obtained through the Obstetrics and Gynecology Tissue Core. Tissues were enzymatically digested and embedded in Matrigel domes (16). Organoids were cultured in ovarian organoid medium and treated with ERX-208. Cell viability was measured using CellTiter-Glo 3D. Images were captured using bright-field microscopy.

### PDX models

All animal studies were carried out using Institutional Animal Care and Use Committee approval from UT Health San Antonio. PDX models (OCa-38 and OCa-67) were generated by implanting fresh human ovarian tumor fragments subcutaneously into immunodeficient SCID mice using an established protocol (18). When PDX tumors reached a volume of 100 to 150 mm^3^, mice were randomized into four treatment groups: vehicle control, ERX-208 (10 mg/kg, administered intraperitoneally 5 days per week), cisplatin (1.5 mg/kg, administered intraperitoneally for a total of three doses), or combination therapy (*n* = 5 tumors per group). For the therapy-resistant OCa-38 PDX model, mice were assigned to two treatment groups: vehicle control or ERX-208 (20 mg/kg, administered intraperitoneally 5 days per week; *n* = 6 tumors per group). Tumor volumes were measured using calipers (volume = ½ × length × width^2^). Tumors were collected at endpoint for weight measurement and downstream analyses.

### Immunohistochemistry (IHC)

IHC staining was performed following a previously published protocol (19). Formalin-fixed, paraffin-embedded tissues were cut into 5-μm sections, deparaffinized, rehydrated, and subjected to antigen retrieval. Sections were blocked in 5% serum and incubated with primary antibodies against Ki-67, GRP78, or γH2AX overnight at 4°C. HRP-conjugated secondary antibodies and 3,3′-Diaminobenzidine (DAB) chromogen were used for detection. Slides were counterstained with hematoxylin, mounted, and imaged. Ki-67–positive nuclei were quantified using ImageJ software. GRP78 and γH2AX expression in IHC sections was also quantified using ImageJ based on H-DAB staining intensity.

### Statistical analysis

Statistical analyses were performed using GraphPad Prism 10. Comparisons between groups were made using the Student *t* test or ANOVA as appropriate. Data are presented as mean ± SEM. Statistical significance was defined as *P* < 0.05.

## Results

### ERX-208 sensitizes ovarian cancer cells to DNA-damaging agents

Our prior studies identified ERX-208 as a potent tris-benzamide with activity against multiple ovarian cancer models (12). To further optimize the clinical translation of ERX-208, we evaluated whether combinations with other agents would enhance ERX-208 activity. Toward this end, we performed an unbiased drug-combination screen in which ERX-208 was combined with a curated library of 147 FDA-approved compounds and assessed for effects on cell viability either alone or in combination using an MTT assay in OCa-39 cells (Fig. 1A). Compounds spanning diverse mechanistic classes were included, enabling systematic identification of drug classes in which activity is potentiated by ERX-208. This screening strategy revealed a strong enrichment of synergistic interactions between ERX-208 and DNA-damaging chemotherapies. To quantitatively assess drug-combination efficacy, synergy was evaluated using Loewe additivity modeling implemented in the SynergyFinder Plus framework. Scatter plot analysis of Loewe synergy scores versus statistical significance (−log_10_*P* value) demonstrated that multiple DNA-damaging agents exhibited both high synergy scores and strong statistical support when combined with ERX-208 (Fig. 1B; Supplementary Fig. S1). Selected synergistic compounds were annotated, highlighting drug classes that most robustly benefit from ERX-208 cotreatment.

### ERX-208 suppresses genome maintenance pathways

Because the original transcriptomic analysis focused on ER stress responses, we reanalyzed the dataset to identify pathways that could explain chemotherapy sensitization. Transcriptomic profiling of ovarian cancer cells treated with ERX-208 revealed a coordinated reprogramming of pathways governing genome maintenance, cell-cycle progression, and stress responses. Gene set enrichment analysis demonstrated strong positive enrichment of apoptotic pathways, consistent with the activation of stress signaling and priming for cell death (Fig. 1C). In contrast, pathways involved in cell cycle progression, and DNA replication, were markedly suppressed following ERX-208 treatment (Fig. 1C). Furthermore, expression levels of RAD51, FANCD2, BRCA1, and CHEK1 were markedly reduced relative to untreated controls. In contrast, ATR expression was modestly increased (Fig. 1D). These findings indicate that ERX-208 selectively downregulates core DNA repair and checkpoint effectors while concomitantly inducing ATR, suggesting a potential rewiring of DDR signaling that may sensitize cells to replication stress. Collectively, these data support a model in which ERX-208 compromises DNA repair capacity and checkpoint control while simultaneously activating stress and apoptotic signaling. This transcriptional state is predicted to increase reliance on residual repair mechanisms and may thereby sensitize ovarian cancer cells to genotoxic chemotherapies.

### LIPA is required for ERX-208–mediated inhibition of ovarian cancer cell survival

To further establish the LIPA dependence of ERX-208 activity, we generated LIPA-KO SKOV3 cells and compared their response with parental SKOV3 cells. Western blot analysis confirmed the loss of LIPA in LIPA-deficient cells, whereas the loading control remained unchanged (Supplementary Fig. S2A). The disappearance of these bands following LIPA depletion supports their identification as endogenous LIPA and adds further validation of antibody specificity. The presence of multiple LIPA species is consistent with different processed and glycosylated forms of the protein. We next examined the functional requirement for LIPA in response to ERX-208. ERX-208 markedly reduced cell viability in parental SKOV3 cells. Conversely, LIPA-KO cells showed reduced sensitivity to ERX-208 (Supplementary Fig. S2B). Consistent with these findings, ERX-208 markedly increased cell death in parental SKOV3 cells. In contrast, LIPA KO significantly reduced ERX-208–induced cell death (Supplementary Fig. S2C). However, LIPA KO cells remained responsive to cisplatin, exhibiting reduced cell viability and increased apoptosis, suggesting that the pathways mediating cisplatin sensitivity remain functional in the absence of LIPA. Together, these genetic and functional studies validate the identity of the LIPA-immunoreactive bands and demonstrate that LIPA is a major determinant of ERX-208 activity in ovarian cancer cells. The reduced sensitivity of LIPA-deficient cells provides independent genetic evidence supporting the LIPA-dependent pharmacologic activity of ERX-208.

### ERX-208 enhances platinum- and taxane-induced cytotoxicity, apoptosis, and invasion suppression

**Figure 2.**
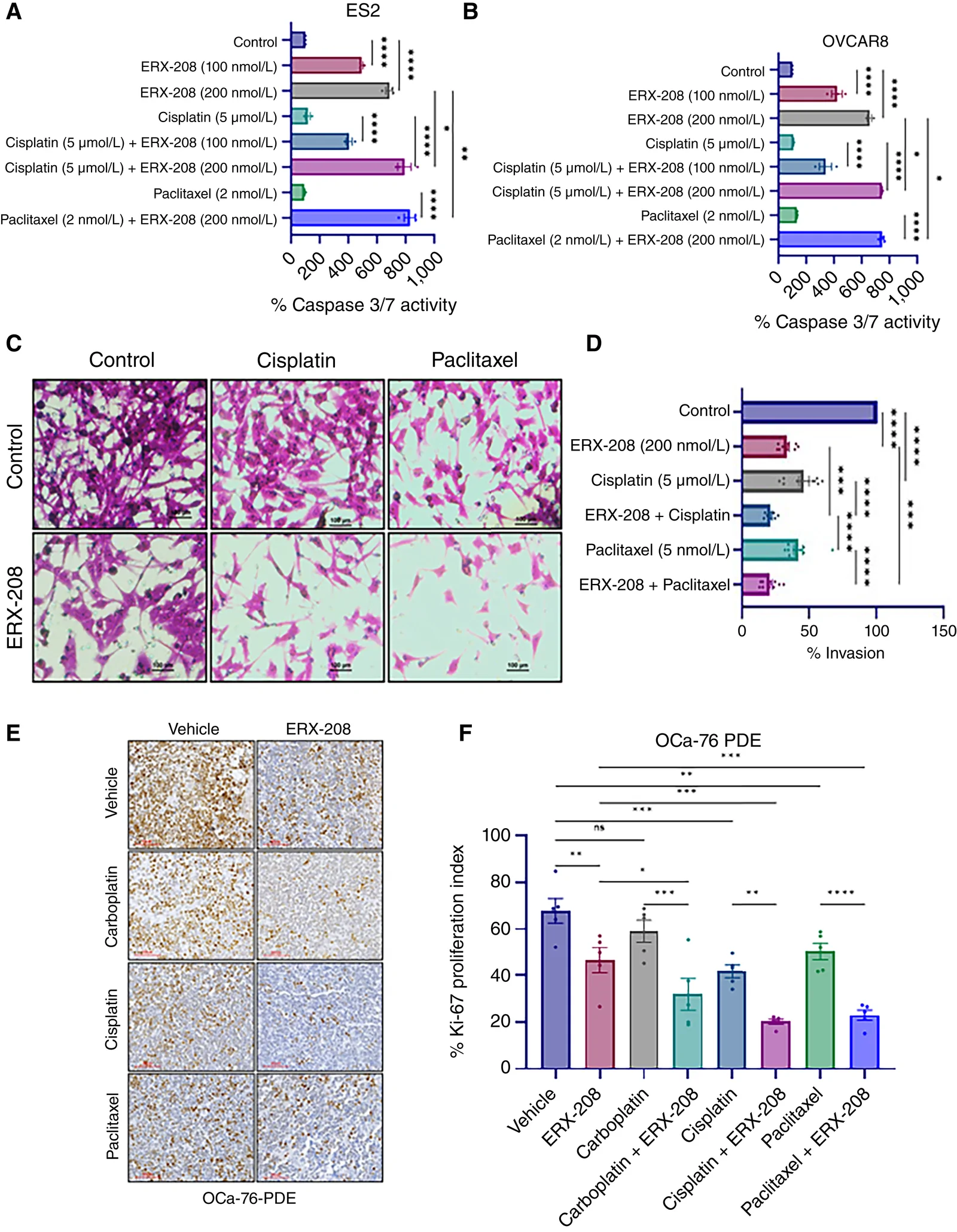
ERX-208 synergizes with platinum- and taxane-based chemotherapies to enhance apoptosis and reduce invasion and proliferation in ovarian cancer models. **A** and **B,** Caspase-3/7 activity in ES2 (**A**) and OVCAR8 (**B**) cells following treatment with ERX-208 (100–200 nmol/L), cisplatin (5 μmol/L), paclitaxel (2 nmol/L), or combinations. ERX-208 markedly enhanced chemotherapy-induced apoptosis. **C** and **D,** Matrigel invasion assay showing reduced invasive capacity of ES2 cells treated with ERX-208 (200 nmol/L), cisplatin (5 μmol/L), paclitaxel (5 nmol/L), or combination treatments. ERX-208 markedly enhanced the anti-invasive effects of cisplatin and paclitaxel. Representative images (**C**) and quantification (**D**) are shown. **E,** Representative IHC staining for Ki-67 in OCa-76-PDE tumor sections following treatment with vehicle, ERX-208, carboplatin, cisplatin, or paclitaxel, either alone or in combination with ERX-208. **F,** Quantification of explant cell proliferation is presented as the percentage of Ki-67–positive cells (Ki-67 proliferation index) for each treatment group. Data show mean ± SEM, with statistical significance indicated as *, *P* < 0.05; **, *P* < 0.01; ***, *P* < 0.001; ****, *P* < 0.0001.

To further study this hypothesis, we used platinum and paclitaxel, two standard-of-care agents in ovarian cancer. In OCa-30, ES2, and OVCAR8 cells, we noted that although ERX-208 reduced cell viability as a single agent, significant synergy was observed with carboplatin, cisplatin, or paclitaxel (Fig. 1E–G). Dose–response analyses in TOV21G, TOV112D, and A2780 cells demonstrated that a fixed low dose of ERX-208 (100 nmol/L) shifted platinum dose–response curves downward, increasing sensitivity to both cisplatin and carboplatin (Supplementary Fig. S3A–S3F). Concordantly, caspase-3/7 activity increased with ERX-208 alone and was further elevated by the combinations (Fig. 2A and B), indicating enhanced apoptosis. Invasion through Matrigel was significantly reduced by ERX-208 and further suppressed when combined with either cisplatin or paclitaxel (Fig. 2C and D).

To assess the impact of ERX-208 and standard chemotherapeutics on tumor cell proliferation, we performed Ki-67 IHC on OCa-76-PDE samples following treatment with vehicle, ERX-208, carboplatin, cisplatin, or paclitaxel, alone and in combination with ERX-208 (Fig. 2E and F). Vehicle-treated explants exhibited abundant Ki-67–positive nuclei, indicating high proliferative activity. ERX-208 monotherapy reduced the proportion of Ki-67–positive cells compared with the vehicle. Chemotherapy alone also decreased Ki-67 staining, with cisplatin and paclitaxel showing a greater reduction in Ki-67 staining than carboplatin. Carboplatin alone caused a modest reduction, which was further enhanced by the combination treatment with ERX-208. Similarly, cisplatin and paclitaxel each markedly reduced Ki-67 positivity, and cotreatment with ERX-208 resulted in additional suppression of proliferative activity.

### ERX-208 enhances DNA damage in combination with platinum and taxane agents and enhances UPR

**Figure 3.**
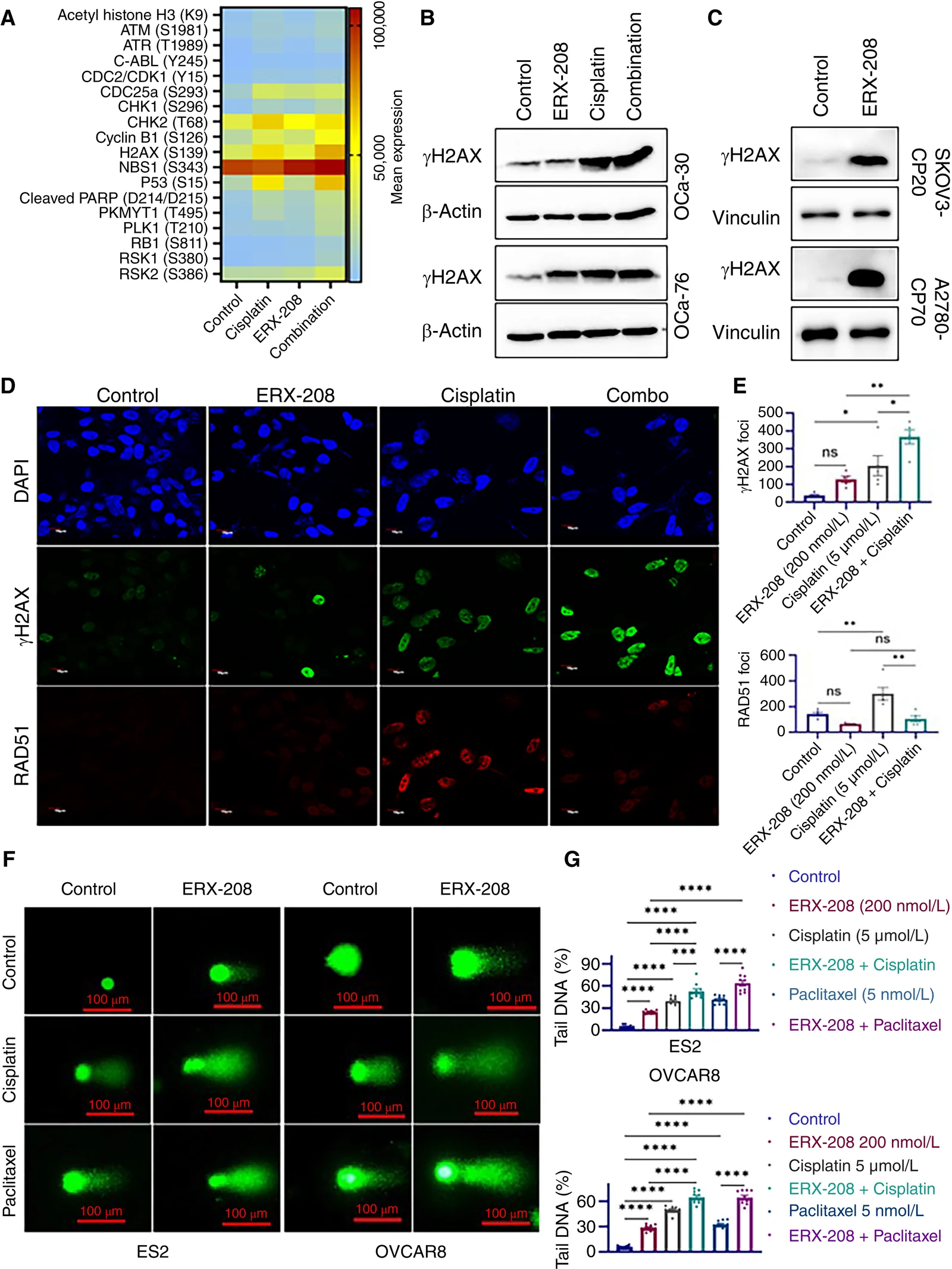
ERX-208 enhances chemotherapy-associated DNA damage and impairs RAD51-mediated DNA repair in ovarian cancer cells. **A,** Heatmap of phospho-protein signaling showing activation of DDR pathways in ES2 cells treated with ERX-208 (200 nmol/L), cisplatin (5 μmol/L), or the combination for 24 hours. Increased phosphorylation of CHK1, CHK2, H2AX, and cleaved PARP indicates enhanced DNA damage signaling, particularly with combination treatment. **B,** Immunoblot analysis of γH2AX expression in patient-derived ovarian cancer cells (OCa-30 and OCa-76) treated with ERX-208 (200 nmol/L), cisplatin (5 μmol/L), or the combination for 24 hours. β-Actin serves as a loading control. Combination treatment shows the strongest induction of γH2AX. **C,** Western blot showing γH2AX induction in therapy-resistant ovarian cancer cell lines (SKOV3-CP20 and A2780-CP70) following treatment with ERX-208 (200 nmol/L). Vinculin serves as a loading control. **D** and **E,** Immunofluorescence detection of γH2AX and RAD51 foci in ES2 cells treated with ERX-208 (200 nmol/L), cisplatin (5 μmol/L), or the combination for 24 hours. Scale bars, 10 μm. **F,** Alkaline comet assay images showing accumulation of alkali-labile DNA lesions and DNA strand breaks in ES2 and OVCAR8 cells treated with ERX-208 (200 nmol/L), cisplatin (5 μmol/L), paclitaxel (5 nmol/L), or combinations for 20 hours. Scale bars, 100 μm. Cells treated with ERX-208 in combination with cisplatin or paclitaxel exhibit markedly increased alkaline comet tail formation. **G,** Quantification of comet tail DNA (%) in ES2 and OVCAR8 cells treated as in **F**. ERX-208 significantly increases DNA damage as a single agent and further augments DNA damage in combination with cisplatin or paclitaxel. Data are presented as mean ± SEM, with statistical significance indicated as *, *P* < 0.05; **, *P* < 0.01;  ***, *P* < 0.001; ****, *P* < 0.0001; ns, not significant. Scale bars, 100 μm.

To delineate the mechanism of chemosensitization, we examined the DNA damage signaling using a phospho-protein array. This analysis revealed robust induction of the DDR following ERX-208 treatment, with further increases following combination with cisplatin. Notably, phosphorylation of ATM/ATR substrates (CHK1 and CHK2), histone H2AX (S139), and PARP cleavage were elevated (Fig. 3A; Supplementary Fig. S4A and S4B). Western blotting confirmed increased γH2AX following ERX-208 ± cisplatin in two patient-derived cell models (OCa-30 and OCa-76; Fig. 3B) and in platinum-resistant SKOV3-CP20 and A2780-CP70 lines (Fig. 3C).

To determine whether ERX-208 alters the DDR to cisplatin, we examined γ-H2AX and RAD51 foci following treatment with ERX-208 alone, cisplatin alone, or the combination. ERX-208 alone produced only a modest increase in γH2AX foci compared with the vehicle control. Cisplatin increased the γH2AX foci, and the ERX-208/cisplatin combination resulted in the greatest accumulation of γH2AX foci, significantly greater than single-agent treatment. The findings suggest that ERX-208 promotes the persistence or accumulation of DNA damage induced by cisplatin. However, the formation of RAD51 foci responded differently. Cisplatin markedly increased RAD51 foci, consistent with the activation of HR repair following DNA damage. ERX-208 alone did not increase RAD51 foci, and importantly, ERX-208 significantly reduced cisplatin-induced RAD51 foci formation. Thus, despite increased γH2AX accumulation, ERX-208–treated cells failed to mount an effective RAD51 response to cisplatin. Together, these data support a model in which ERX-208 sensitizes ovarian cancer cells to cisplatin by increasing DNA damage and inhibiting RAD51-dependent HR repair. The combination of increased DNA damage and defective repair gives a mechanistic basis for the increased therapeutic activity of ERX-208 in combination with platinum-based chemotherapy (Fig. 3D and E).

To further characterize the effect of ERX-208 on chemotherapy-induced DNA damage, we performed alkaline comet assays in ES2 and OVCAR8 ovarian cancer cells treated with ERX-208 alone or in combination with cisplatin or paclitaxel. Consistent with the scope of this assay, increased comet tail DNA was interpreted as the accumulation of alkali-labile DNA lesions and DNA strand breaks rather than as a specific measure of DNA double-strand breaks. ERX-208 treatment significantly increased the percentage of tail DNA compared with vehicle-treated cells in both ES2 and OVCAR8 cells. As expected, cisplatin and paclitaxel also increased DNA damage. Importantly, combining ERX-208 with either cisplatin or paclitaxel resulted in significantly greater tail DNA than ERX-208 or the respective chemotherapy agent alone (Fig. 3F and G). These results demonstrate that ERX-208 enhances the accumulation of chemotherapy-associated DNA lesions in ovarian cancer cells.

Because the alkaline comet assay alone does not distinguish increased initial DNA injury from defective DNA damage resolution, these findings were complemented by drug washout and time-course studies assessing the persistence and resolution of DNA damage. DNA damage signaling was assessed by Western blot analysis of γH2AX and RAD51, a marker of homologous recombination repair (Supplementary Fig. S4C and S4D). Drug exposure increased γH2AX levels that persisted during the recovery period in ERX-208–treated cells. Conversely, RAD51 showed a unique temporal profile of induction after DNA damage and a marked decrease during recovery in the presence of ERX-208. Persistent γH2AX during the recovery period was consistent with delayed resolution of chemotherapy-induced DNA damage, coincident with reduced RAD51 levels in ERX-208–treated cells. These washout experiments complement the alkaline comet assay results and provide temporal evidence that the increased DNA damage observed with ERX-208 correlates with prolonged DNA damage signaling rather than simply reflecting greater initial injury. These results are consistent with an inhibitory effect of ERX-208 on the efficient resolution of chemotherapy-induced DNA damage, in conjunction with the diminished RAD51 response. Together, the comet assay and damage resolution studies support the conclusion that ERX-208 increases chemotherapy-associated DNA damage and delays its resolution.

Given the prominent UPR signature, we profiled key pathway components under single-agent and combination treatment conditions. In A2780 cells, ERX-208 increased the expression of key ER stress–associated genes including HSPA5/BiP, XBP1, PERK, and CHOP. Cisplatin alone induced the activation of a subset of these genes, whereas the combination treatment resulted in the most robust transcriptional activation (Supplementary Fig. S5A). Similar expression patterns were observed with paclitaxel cotreatment (Supplementary Fig. S5B). At the mRNA level, ERX-208 promoted sXBP1, as detected by RT-PCR, and cotreatment with cisplatin further increased sXBP1 abundance (Supplementary Fig. S5C). ERX-208 also enhanced phosphorylation of eIF2α, a hallmark of PERK activation, both alone and in combination with cisplatin or paclitaxel (Supplementary Fig. S5D and S5E). Collectively, these results suggest that ERX-208 potentiates platinum- and taxane-based chemotherapy by concurrently amplifying DNA damage signaling and activating UPR pathways, thereby overwhelming tumor stress tolerance and repair capacity to enhance cancer cell killing effect.

### ERX-208 reduces viability and clonogenicity of therapy-resistant ovarian cancer cells and activates ER stress signaling

**Figure 4.**
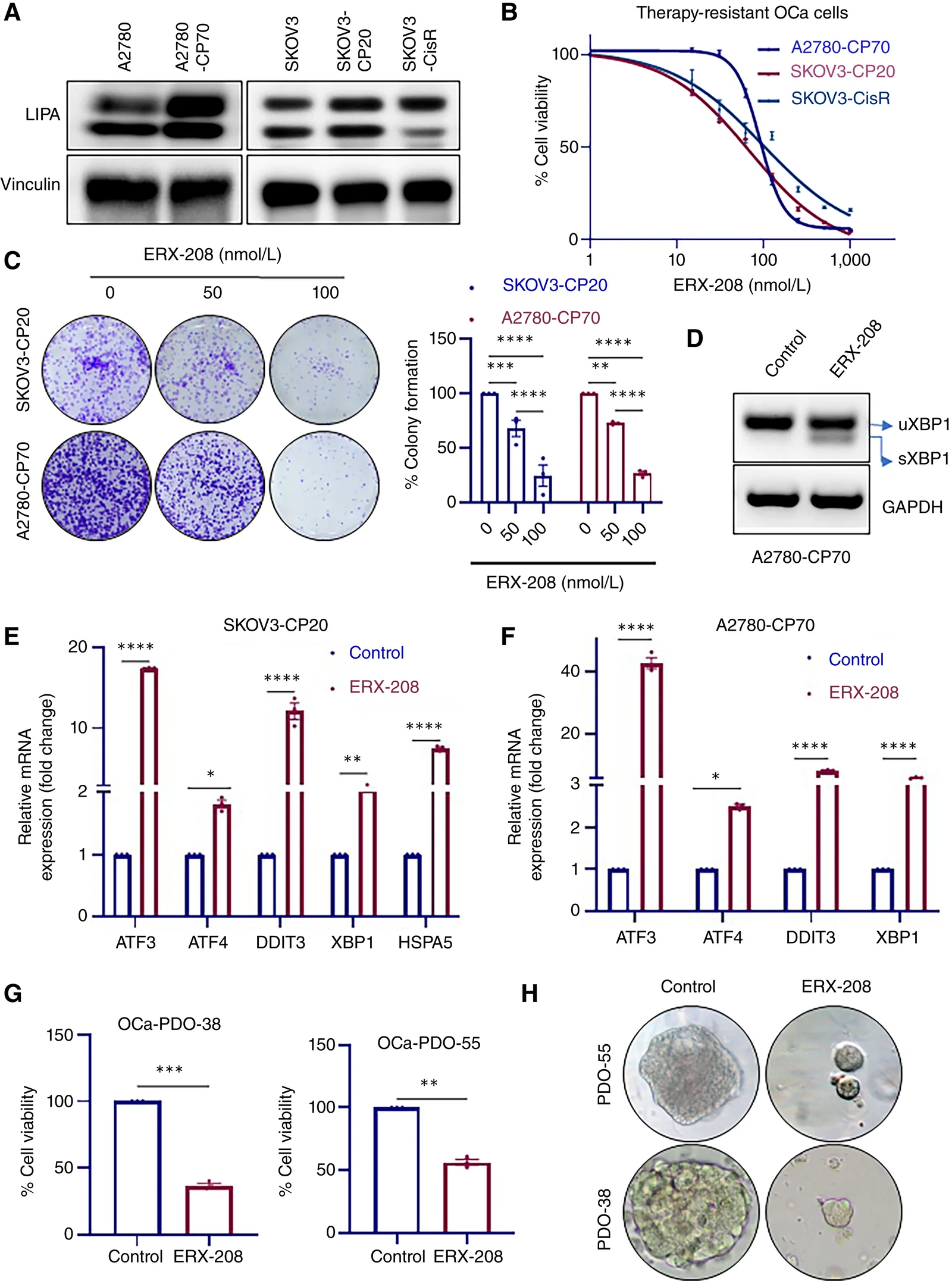
ERX-208 inhibits viability and induces ER stress in therapy-resistant ovarian cancer (OCa) models. **A,** Western blot analysis of LIPA protein expression in parental (A2780 and SKOV3) and therapy-resistant ovarian cancer cell lines (A2780-CP70, SKOV3-CP20, and SKOV3-CisR). Vinculin served as the loading control. **B,** Dose–response curves showing decreased cell viability in therapy-resistant ovarian cancer cell lines (A2780-CP70, SKOV3-CP20, and SKOV3-CisR) treated with increasing concentrations of ERX-208 for 72 hours. Viability was assessed using MTT cell viability assay. **C,** Clonogenic survival assay of A2780-CP70 and SKOV3-CP20 cells treated with ERX-208 (50–100 nmol/L). Colonies were fixed, stained, and quantified after 10 to 14 days, demonstrating dose-dependent inhibition of long-term survival. Quantification of the percentage of colonies was shown as a bar graph. **D,** RT-PCR analysis of XBP1 splicing in A2780-CP70 cells treated with ERX-208. The appearance of sXBP1 indicates ER stress activation. GAPDH was used as a loading control. **E** and **F,** qRT-PCR analysis of ER stress–related genes (ATF3, ATF4, DDIT3/CHOP, XBP1, and HSPA5/BiP) in SKOV3-CP20 (**E**) and A2780-CP70 (**F**) cells treated with ERX-208 for 24 hours. **G,** Cell viability of therapy-resistant patient-derived ovarian cancer organoids (PDO-38 and PDO-55) treated with ERX-208. ERX-208 significantly reduced viability compared with control organoids. **H,** Representative bright-field images of PDO-38 and PDO-55 organoids treated with ERX-208, showing reduced organoid size and structural integrity compared with controls. Data are presented as mean ± SEM, with statistical significance indicated as  *, *P* < 0.05; **, *P* < 0.01; ***, *P* < 0.001; ****, *P* < 0.0001; ns, not significant.

We then assessed the antitumor activity of ERX-208 in ovarian cancer models with acquired chemoresistance. LIPA protein, previously linked to aggressive phenotypes, was readily detected in therapy-resistant derivatives A2780-CP70, SKOV3-CP20, and SKOV3-CisR (Fig. 4A). ERX-208 exposure decreased viability across all resistant lines in a dose-dependent manner (Fig. 4B) and significantly suppressed long-term survival in clonogenic assays (Fig. 4C). Mechanistically, ERX-208 induced robust splicing of XBP1 (sXBP1) in A2780-CP70 cells (Fig. 4D), consistent with activation of the IRE1α arm of the UPR. qRT-PCR confirmed the upregulation of canonical ER stress/UPR genes, *ATF3*, *ATF4*, *DDIT3*/*CHOP*, and *XBP1* in both SKOV3-CP20 and A2780-CP70 cells (Fig. 4E and F). Importantly, ERX-208 reduced the viability of therapy-resistant ovarian cancer PDOs (PDO-38 and PDO-55; Fig. 4G) and disrupted organoid architecture (Fig. 4H), highlighting translational relevance.

### ERX-208 suppresses tumor growth in therapy-resistant ovarian cancer PDX models and enhances platinum efficacy *in vivo*

**Figure 5.**
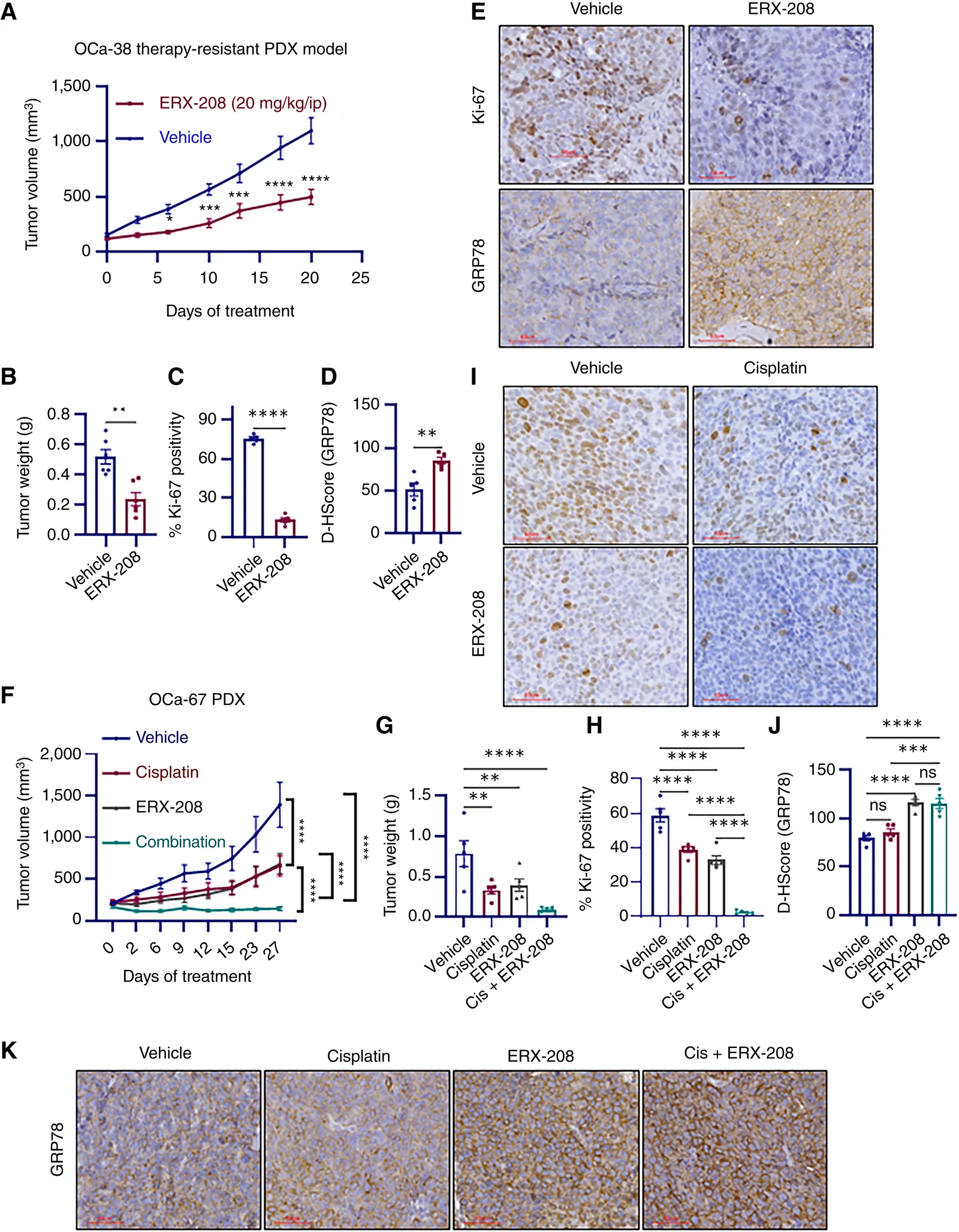
ERX-208 suppresses tumor growth in ovarian cancer PDX models and enhances response to cisplatin. **A,** Tumor growth curves of the therapy-resistant OCa-38 PDX model treated with vehicle or ERX-208 (20 mg/kg, intraperitoneally). ERX-208 significantly reduced tumor growth during treatment. **B,** Final tumor weights from OCa-38 PDX mice treated with vehicle or ERX-208. ERX-208 markedly decreased tumor weight at the study endpoint. **C,** Ki-67 quantification in response to ERX-208 treatment. **D,** GRP78 quantification of vehicle and ERX-208 treatments. **E,** Representative IHC staining for Ki-67 and GRP78 in OCa-38 PDX tumors treated with vehicle and ERX-208. **F,** Tumor volume measurements in OCa-67 PDX mice treated with vehicle, cisplatin (1.5 mg/kg), ERX-208 (10 mg/kg), or the combination. Combination therapy produced the strongest tumor growth suppression, with near-complete inhibition of tumor progression. **G,** Final tumor weights corresponding to **F**. **H,** Ki-67 quantification in response to treatments. **I,** Representative IHC staining for Ki-67 in OCa-67 PDX tumors treated with vehicle, cisplatin, ERX-208, or combination. **J,** GRP78 quantification in response to treatments. **K,** Representative IHC staining for GRP78 in OCa-67 PDX tumors treated with vehicle, cisplatin, ERX-208, or combination. Data are presented as mean ± SEM, with statistical significance determined by two-way ANOVA; *, *P* < 0.05; **, *P* < 0.01; ***, *P* < 0.001; ****, *P* < 0.0001; ns, not significant.

To evaluate the *in vivo* antitumor activity of ERX-208, we first examined its efficacy in a therapy-resistant ovarian cancer PDX model (OCa-38). Treatment with ERX-208 (20 mg/kg, intraperitoneally) resulted in a significant reduction in tumor growth compared with vehicle control over the course of treatment (Fig. 5A). Consistent with the observed tumor growth inhibition, ERX-208–treated mice exhibited significantly lower terminal tumor weights relative to vehicle-treated controls (Fig. 5B). IHC analyses of OCa-38 PDX tumors revealed that ERX-208 treatment markedly reduced tumor cell proliferation, as evidenced by a significant decrease in Ki-67–positive cells (Fig. 5C and E). In parallel, ERX-208 significantly increased GRP78 staining intensity compared with vehicle treatment (Fig. 5D and E). Furthermore, ERX-208 induced DNA damage in OCa-38 PDX tumors, as assessed by γH2AX immunostaining (Supplementary Fig. S6A and S6B).

We next assessed whether ERX-208 could enhance the antitumor efficacy of platinum chemotherapy using the OCa-67 PDX model. Although cisplatin or ERX-208 monotherapy each modestly suppressed tumor growth, the combination of ERX-208 with cisplatin produced a significantly greater inhibition of tumor volume compared with either agent alone (Fig. 5F). This enhanced antitumor effect was accompanied by a pronounced reduction in terminal tumor weight in the combination group relative to vehicle and single-agent treatments (Fig. 5G). IHC analyses of OCa-67 tumors demonstrated that combination treatment resulted in a marked decrease in tumor cell proliferation, with significantly fewer Ki-67–positive cells compared with cisplatin or ERX-208 monotherapy (Fig. 5H and I). In contrast, GRP78 expression was elevated in ERX-208–treated tumors, both as a single agent and in combination with cisplatin, indicating sustained pathway engagement in the combination setting (Fig. 5J and K). Therapy-induced DNA damage was further confirmed in tumor tissues by γH2AX IHC. ERX-208 increased γH2AX staining compared with control tumors, whereas chemotherapy also increased γH2AX levels. The combination treatment produced the highest γH2AX signal, supporting enhanced accumulation of DNA damage *in vivo* following ERX-208 plus chemotherapy (Supplementary Fig. S6C and S6D). Collectively, these *in vivo* data demonstrate that ERX-208 exerts potent antitumor activity in therapy-resistant ovarian cancer PDX models, suppresses tumor cell proliferation, and significantly enhances the efficacy of platinum chemotherapy. The combination-induced tumor growth inhibition and reduced proliferative index support a model in which ERX-208 sensitizes ovarian cancer tumors to platinum-based treatment through sustained modulation of ER stress and survival pathways.

## Discussion

Currently approved therapies (such as cytoreductive surgery, platinum-based chemotherapy, and PARP inhibitors) have improved ovarian cancer survival for clinically localized disease (1). However, current therapies have limited efficacy after disease relapse, and survival rates remain poor for patients with advanced ovarian cancer. Chemotherapy has been shown to induce ER stress by disrupting protein-folding homeostasis and activating the UPR (20). Depending on the cellular context, this ER stress may promote apoptosis or contribute to chemoresistance (21). In ovarian cancer chemotherapy, cisplatin has particularly been shown to induce ER stress and activate the UPR, contributing to chemoresistance through adaptive pathways such as autophagy (22, 23). Ovarian cancer cells already have high protein synthesis and folding demands, creating substantial basal ER stress that is further amplified by tumor progression, a hostile microenvironment, and chemotherapy (24).

In the present study, using an unbiased screen of FDA-approved agents, we identified a strong enrichment of synergistic interactions between ERX-208 and diverse classes of chemotherapeutics, including platinum agents, anthracyclines, and taxanes. Importantly, as ERX-class compounds are currently undergoing phase I clinical evaluation, the identification of rational combination strategies is particularly relevant for future clinical development. The present study uses a new biological interpretation of previously generated transcriptomic data to generate and test a distinct hypothesis about DNA repair suppression and chemotherapy sensitization. These analyses showed that ERX-208 broadly reprograms cellular pathways by activating stress and apoptotic signaling while suppressing DNA repair, replication, and checkpoint pathways, thereby reducing tumor cells’ tolerance to genotoxic stress. Mechanistically, our data indicate that ERX-208 induces ER stress and amplifies chemotherapy-induced DNA damage while simultaneously impairing the cellular capacity for repair. Increased γH2AX accumulation, reduced RAD51 foci, activation of ATM/ATR signaling, and increased DNA strand breaks detected by alkaline comet assays collectively support enhanced genotoxic stress following ERX-208 treatment. Our drug-washout resolution kinetics provide direct, orthogonal functional evidence that ERX-208 causes a persistent homologous recombination/repair defect rather than merely correlating with reduced RAD51 transcript/protein levels. This finding is also consistent with prior reports that ER stress can stimulate proteasomal degradation of RAD51 and impair double-strand break repair (25), lending independent precedent to the mechanism proposed here. Collectively, our findings amplify the unappreciated link between induced ER stress and impaired genome maintenance.

Our results are consistent with emerging evidence that ER stress can modulate genome stability and DNA repair capacity. Chronic PERK-eIF2α-ATF4–driven UPR signaling has been shown to suppress genome maintenance programs while promoting stress adaptation (22). Chronic ER stress suppresses key DDR components, compromising homologous recombination and replication fork integrity (6). Persistent ER stress activates CHOP, shifting the balance from adaptive signaling toward apoptosis through prodeath transcriptional programs and reduced survival signaling (26). ER stress also intersects with the DDR, and recent work shows that sustained ER stress rapidly decreases DNA-PKcs activity and allows DNA damage to accumulate, facilitating a switch from adaptation to cell death (27). Although the precise molecular cross-talk between these pathways warrants further investigation, our finding strongly suggests that further induction of ER stress by ERX-208 can be used to sensitize ovarian cancer to DNA-damaging agents.

With our studies in clinically applicable regimens, we demonstrate that ERX-208 robustly enhances the cytotoxic effects of platinum and taxane therapies across multiple ovarian cancer models. Combination treatment significantly reduced cell viability, clonogenic survival, and invasive capacity while increasing apoptotic signaling. Importantly, ERX-208 shifted dose–response curves for platinum agents, indicating increased drug sensitivity even at lower concentrations. Furthermore, the therapeutic relevance of our findings is supported by robust validation in clinically representative models, including PDO, PDE, and PDX. ERX-208 demonstrated single-agent activity in therapy-resistant models and significantly enhanced the efficacy of cisplatin *in vivo*. The ability of ERX-208 to overcome resistance in these models is particularly notable, as platinum-resistant ovarian cancer remains a major clinical challenge with limited treatment options. Our data suggest that ERX-208 may bypass traditional resistance mechanisms by simultaneously disrupting protein homeostasis and DNA repair pathways.

Importantly, the antitumor activity of ERX-208 does not require ovarian cancer cells to exhibit constitutive genetic dependence on LIPA. Rather, prior studies demonstrated that LIPA is required for ERX-mediated ER stress signaling and apoptosis. Genetic dependency and pharmacologic target engagement are distinct biological phenomena and need not coincide. These findings also raise the possibility of biomarker-driven clinical translation. The transcriptional suppression of DDR pathways and induction of ER stress markers could serve as pharmacodynamic indicators of response. In addition, tumors with preexisting deficiencies in DNA repair pathways may be particularly sensitive to ERX-208–based combinations, suggesting potential synergy with established DDR-targeting strategies. Future studies should explore the relationship between LIPA expression, ER stress signaling, and genomic instability to identify patient populations most likely to benefit from this approach. Although our study provides strong preclinical evidence, several questions remain. The precise molecular mechanisms by which LIPA inhibition leads to the repression of DDR pathways are not fully defined and may involve alterations in lipid metabolism, membrane composition, or signaling networks that influence chromatin dynamics and repair complex assembly. Finally, the integration of ERX-208 with other targeted therapies, including PARP inhibitors or immune checkpoint blockade, represents an important area for future investigation.

In summary, our study demonstrates that ERX-208 induces a DNA repair–deficient state and sensitizes ovarian cancer to DNA-damaging chemotherapy. By coupling ER stress activation with the suppression of genome maintenance pathways, ERX-208 potentiates chemotherapy-induced cell death and overcomes resistance in clinically relevant models. These findings provide a strong rationale for the clinical development of ERX-208–based combination therapies and support further exploration of ER stress-DDR cross-talk as a therapeutic axis in ovarian cancer.

### Clinical impact

This study identifies the clinical utility of ERX-208 to enhance the efficacy of chemotherapies in ovarian cancer. By inducing sustained ER stress and a functional DNA repair–deficient state, ERX-208 sensitizes both treatment-naïve and therapy-resistant tumors to platinum and taxane agents across multiple clinically relevant models. The present findings have immediate implications for the clinical development of ERX-class compounds. The identification of platinum and taxane therapies as synergistic partners provides a rational framework for future combination studies. Because platinum-based regimens remain the backbone of ovarian cancer therapy, these findings may facilitate the rapid clinical translation of ERX-based combinations. This approach may allow dose reduction, overcome chemotherapy resistance, and expand therapeutic options, with ER stress and DDR markers supporting biomarker-guided patient selection.

## Supplementary Material

Supplementary Figure S1 Figure S1. Loewe synergy analysis of ERX-208 in combination with FDA compounds.

Supplementary Figure S2 Figure S2. LIPA knockout (KO) makes SKOV3 cells resistant to ERX-208 and attenuated ERX-208-induced cell death.

Supplementary Figure S3 Figure S3. ERX-208 enhances the cytotoxic effects of platinum and taxane therapies in additional ovarian cancer models.

Supplementary Figure S4 Figure S4. ERX-208 enhances DNA damage.

Supplementary Figure S5 Figure S5. ERX-208 activates UPR signaling and enhances the anti-proliferative effects of chemotherapy in OCa cells.

Supplementary Figure S6 Figure S6. Increased expression of γH2A.X in ERX-208 treated group compared with vehicle control group in OCa-38 PDX model.

## Data Availability

All data supporting the conclusions of this study are included in the article and/or the Supplementary Materials.
