# Supplementary material for: ERX-208 Induces Endoplasmic Reticulum Stress and Suppresses DNA Damage Repair to Enhance Chemotherapy Sensitivity
Source: Cancer Res Commun. 2026 Oct 29;6(10):2541–54. doi: 10.1158/2767-9764.CRC-26-0421 (PMC13718282; doi:10.1158/2767-9764.CRC-26-0421)
Supplement: Supplementary file 1 [file crc-26-0421_supplementary_figure_s1_suppsf1.pdf]

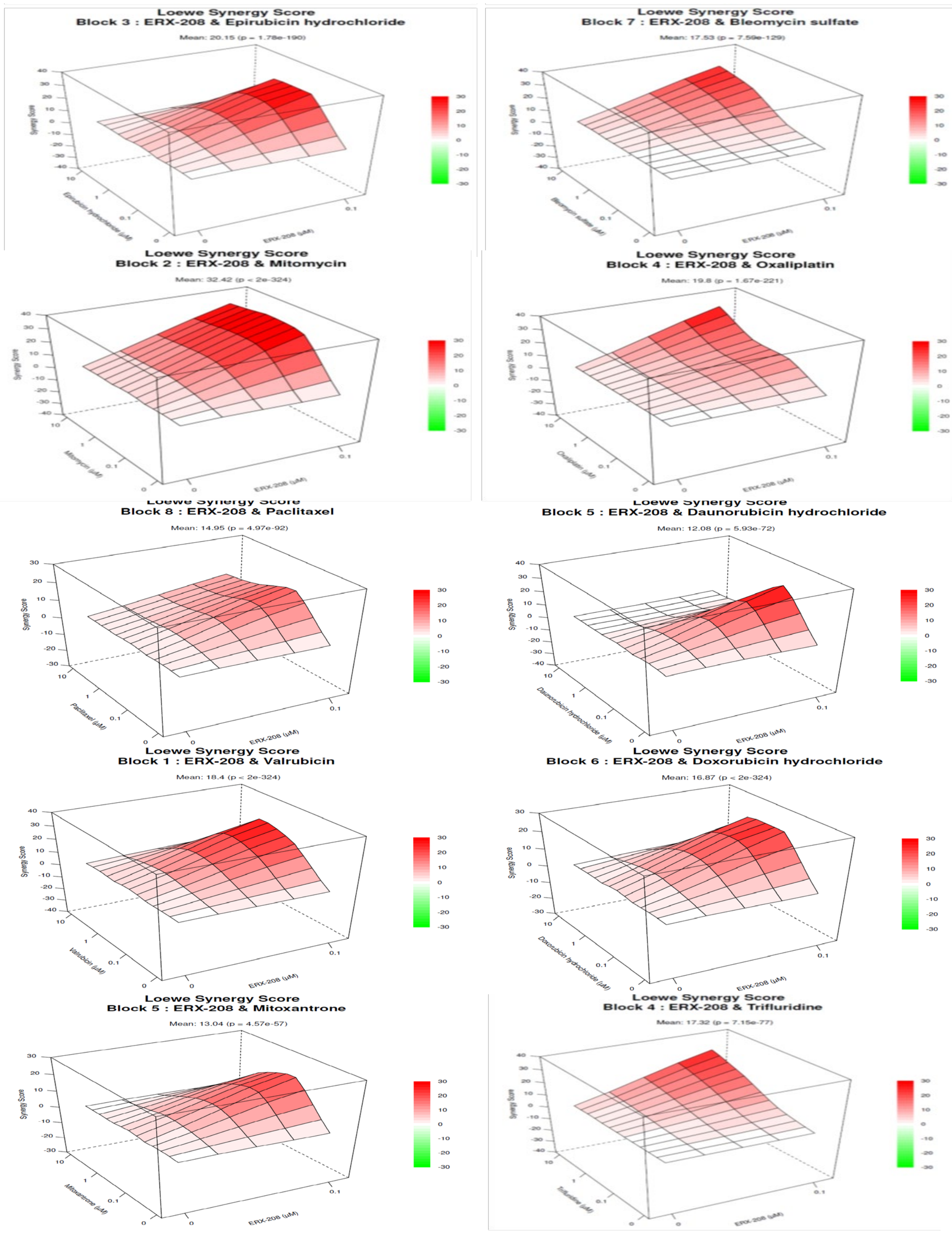

Supplementary Fig. 1. Loewe synergy analysis of ERX-208 in combination with FDA compounds. Three-dimensional response surface plots depict Loewe synergy scores for ERX-208 combined with multiple chemotherapeutic agents, including epirubicin hydrochloride, bleomycin sulfate, mitomycin C, oxaliplatin, paclitaxel, daunorubicin hydrochloride, valrubicin, doxorubicin hydrochloride, mitoxantrone, and trifluridine. ERX-208 concentration (μM) and partner drug concentration (μM) are shown on the x- and y-axes, respectively, while the z-axis represents the calculated Loewe synergy score. Color gradients indicate the degree of drug interaction, with red regions denoting synergistic interactions (positive Loewe scores), white representing additive effects, and green indicating antagonism. Mean Loewe synergy scores for each drug combination are indicated above individual plots. Synergy scores were calculated using Loewe additivity modeling based on cell viability measurements obtained from dose–response matrices.
