# Supplementary material for: ERX-208 Induces Endoplasmic Reticulum Stress and Suppresses DNA Damage Repair to Enhance Chemotherapy Sensitivity
Source: Cancer Res Commun. 2026 Oct 29;6(10):2541–54. doi: 10.1158/2767-9764.CRC-26-0421 (PMC13718282; doi:10.1158/2767-9764.CRC-26-0421)
Supplement: Supplementary file 2 [file crc-26-0421_supplementary_figure_s2_suppsf2.pdf]

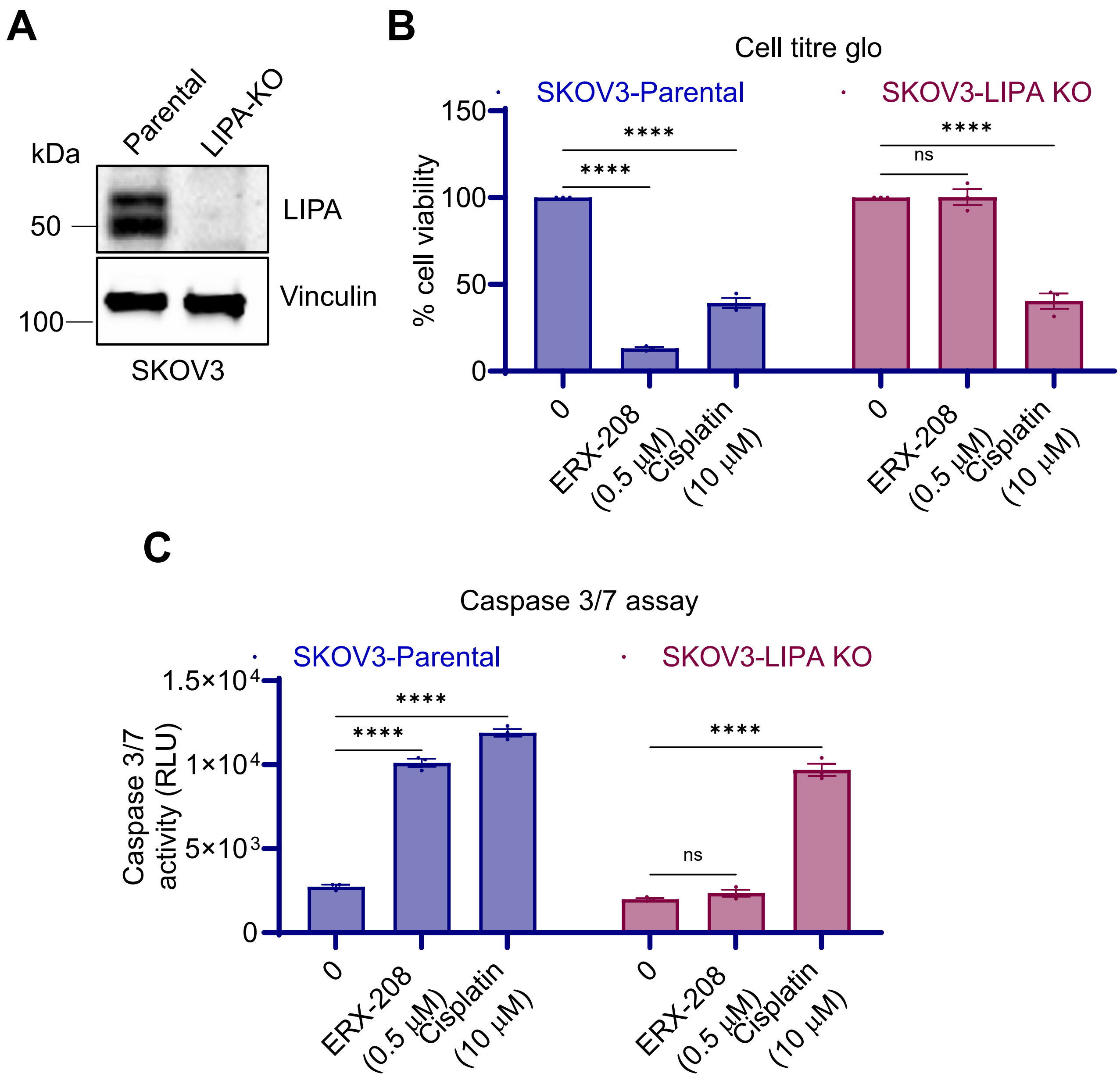

Supplementary Fig. 2. LIPA knockout (KO) makes SKOV3 cells resistant to ERX-208 and attenuated ERX-208-induced cell death (A) Representative Western blot confirming successful knockout of LIPA in SKOV3-LIPA KO cells compared with SKOV3 parental cells. (B) Cell viability analysis of SKOV3 parental and LIPA-KO cells following treatment with vehicle, cisplatin, or ERX-208. LIPA deficiency significantly enhanced the reduction in cell viability induced by cisplatin, whereas ERX-208 treatment produced comparable effects in parental and LIPA-KO cells. (C) Caspase-3/7 activity assay in SKOV3 parental and LIPA-KO cells. LIPA knockout significantly attenuated ERX-208-induced caspase-3/7 activation compared with parental cells, while cisplatin retained apoptotic activity in LIPA-KO cellsLIPA knockout significantly attenuated ERX-208-induced caspase-3/7 activation compared with parental cells, while cisplatin retained apoptotic activity in LIPA-KO cells. These findings support the specificity of ERX-208. Data are presented as mean ± SEM. ****$p < 0.0001$; ns, not significant.
