# Supplementary material for: ERX-208 Induces Endoplasmic Reticulum Stress and Suppresses DNA Damage Repair to Enhance Chemotherapy Sensitivity
Source: Cancer Res Commun. 2026 Oct 29;6(10):2541–54. doi: 10.1158/2767-9764.CRC-26-0421 (PMC13718282; doi:10.1158/2767-9764.CRC-26-0421)
Supplement: Supplementary file 3 [file crc-26-0421_supplementary_figure_s3_suppsf3.pdf]

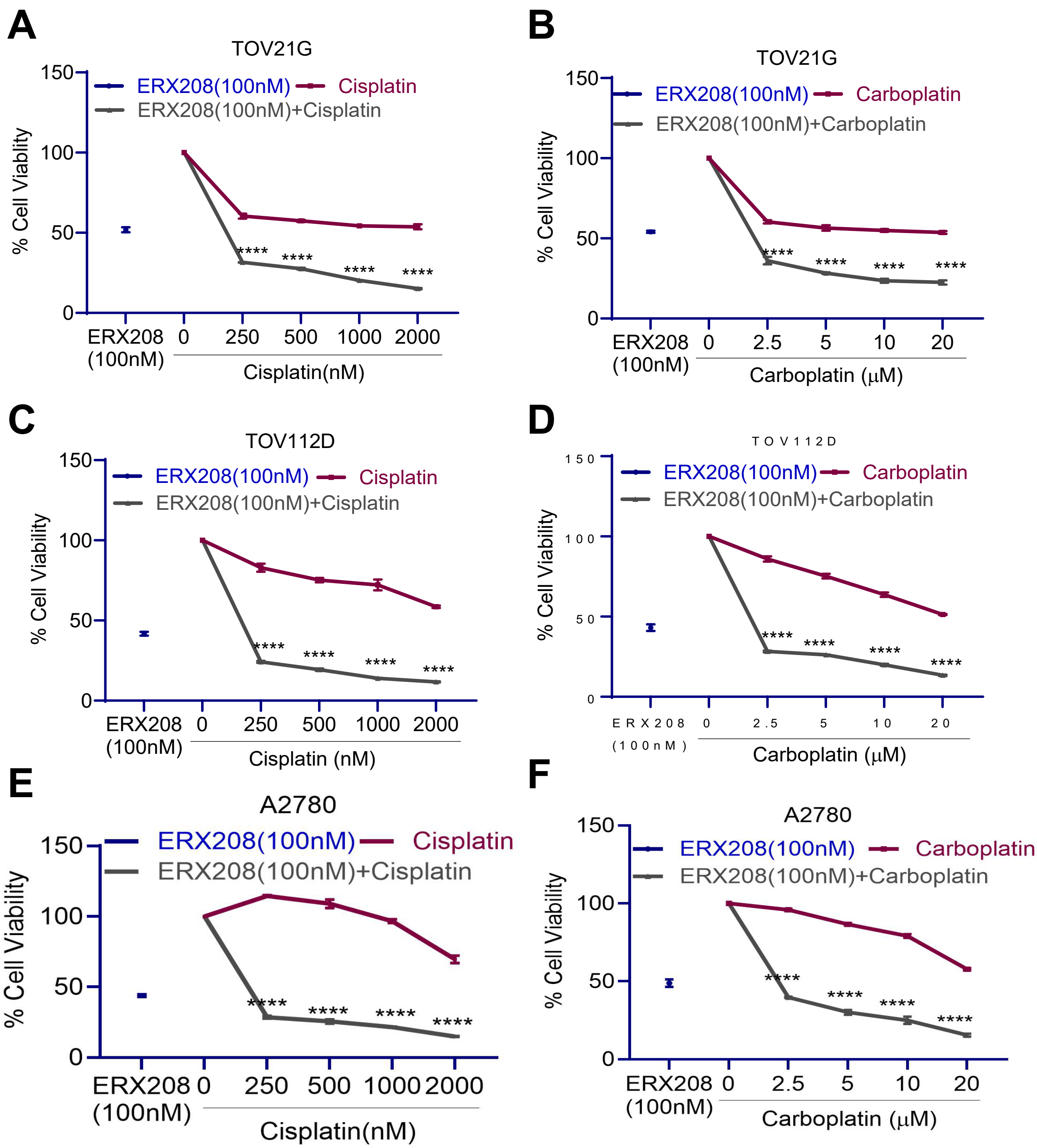

Supplementary Fig. 3. ERX-208 enhances the cytotoxic effects of platinum and taxane therapies in additional ovarian cancer models (A–B) Dose–response curves showing viability of TOV21G cells treated with increasing concentrations of cisplatin (A) or carboplatin (B), alone or in combination with ERX-208 (100 nM) for 72 h. ERX-208 significantly sensitized TOV21G cells to both agents across doses. (C–D) Viability of TOV112D cells treated with increasing concentrations of cisplatin (C) or carboplatin (D), alone or combined with ERX-208 (100 nM) for 72 h. ERX-208 enhanced the cytotoxic effects of both platinum drugs and reduced viability compared with single-agent treatments. (E) Dose–response curves showing that ERX-208 (100 nM) potentiates the cytotoxic effects of cisplatin (0–2000 nM) in A2780 cells. (F) Viability of A2780 cells treated with increasing carboplatin concentrations in the presence or absence of ERX-208 (100 nM). ERX-208 significantly sensitized cells to carboplatin across doses. Data represent mean ± SEM. Statistical significance: ****$p < 0.0001$.
