# Supplementary material for: ERX-208 Induces Endoplasmic Reticulum Stress and Suppresses DNA Damage Repair to Enhance Chemotherapy Sensitivity
Source: Cancer Res Commun. 2026 Oct 29;6(10):2541–54. doi: 10.1158/2767-9764.CRC-26-0421 (PMC13718282; doi:10.1158/2767-9764.CRC-26-0421)
Supplement: Supplementary file 4 [file crc-26-0421_supplementary_figure_s4_suppsf4.pdf]

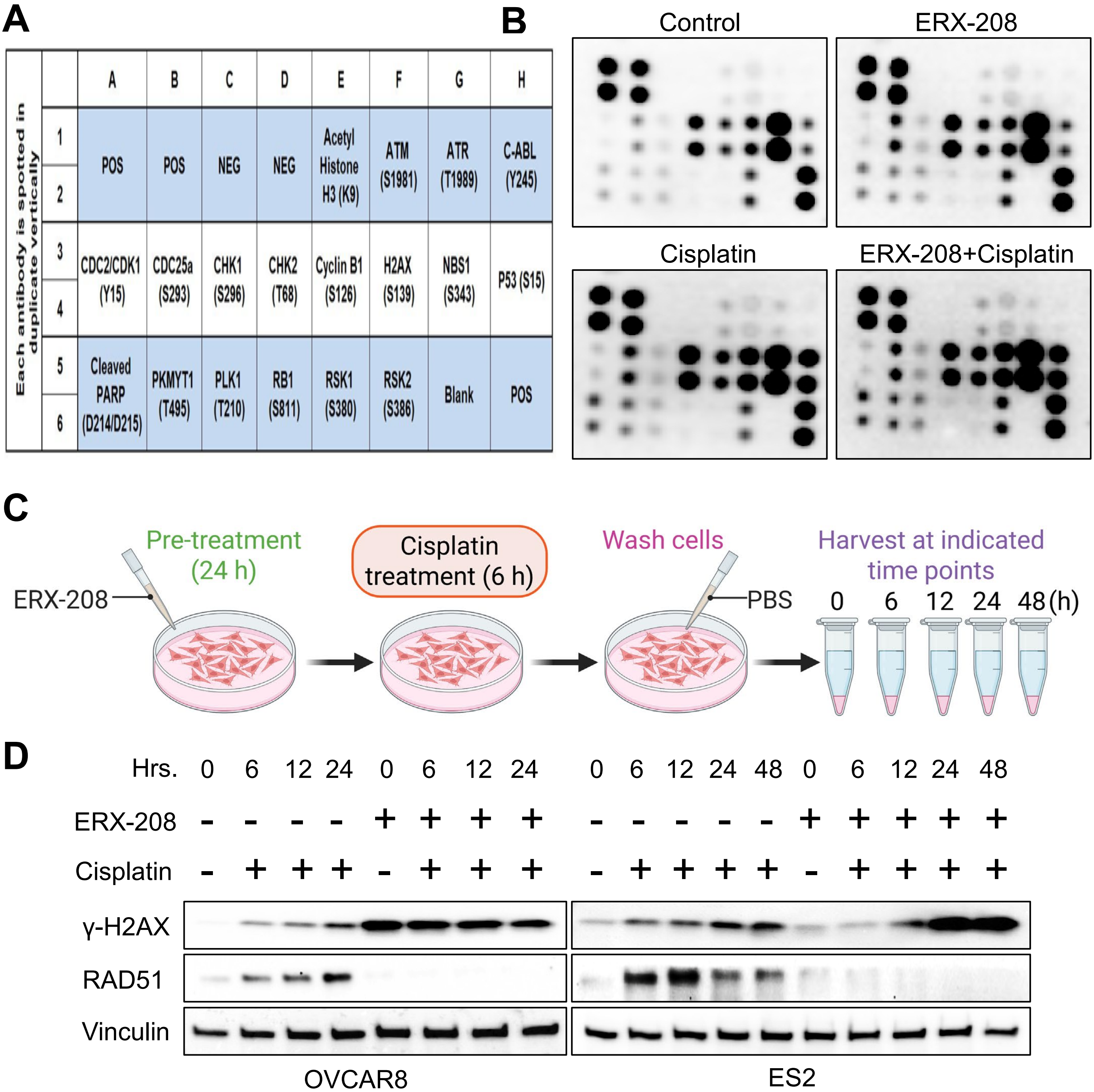

Supplementary Fig. 4. ERX-208 enhances DNA damage. (A-B) phospho-proteomics array map (A) and images of dot blot (B). C-D, Time-dependent effects of cisplatin on DNA damage response proteins in ERX-208 pre-treated OCa cells. (C) Schematic representation of the treatment regimen. OVCAR8 and ES2 cells were pre-treated with ERX-208 (200 nM) for 24 hrs and then exposed to cisplatin (10 μM) for 6 h, washed, and cells were harvested for the indicated times (0, 6, 12, 24h for ES2 and 0, 6, 12, 24, 48h for ES2). (D) Representative Western blots demonstrate temporal changes in DNA damage and homologous recombination repair markers following cisplatin treatment in ERX-208-pre-treated OCa cells.
