# Supplementary material for: ERX-208 Induces Endoplasmic Reticulum Stress and Suppresses DNA Damage Repair to Enhance Chemotherapy Sensitivity
Source: Cancer Res Commun. 2026 Oct 29;6(10):2541–54. doi: 10.1158/2767-9764.CRC-26-0421 (PMC13718282; doi:10.1158/2767-9764.CRC-26-0421)
Supplement: Supplementary file 5 [file crc-26-0421_supplementary_figure_s5_suppsf5.pdf]

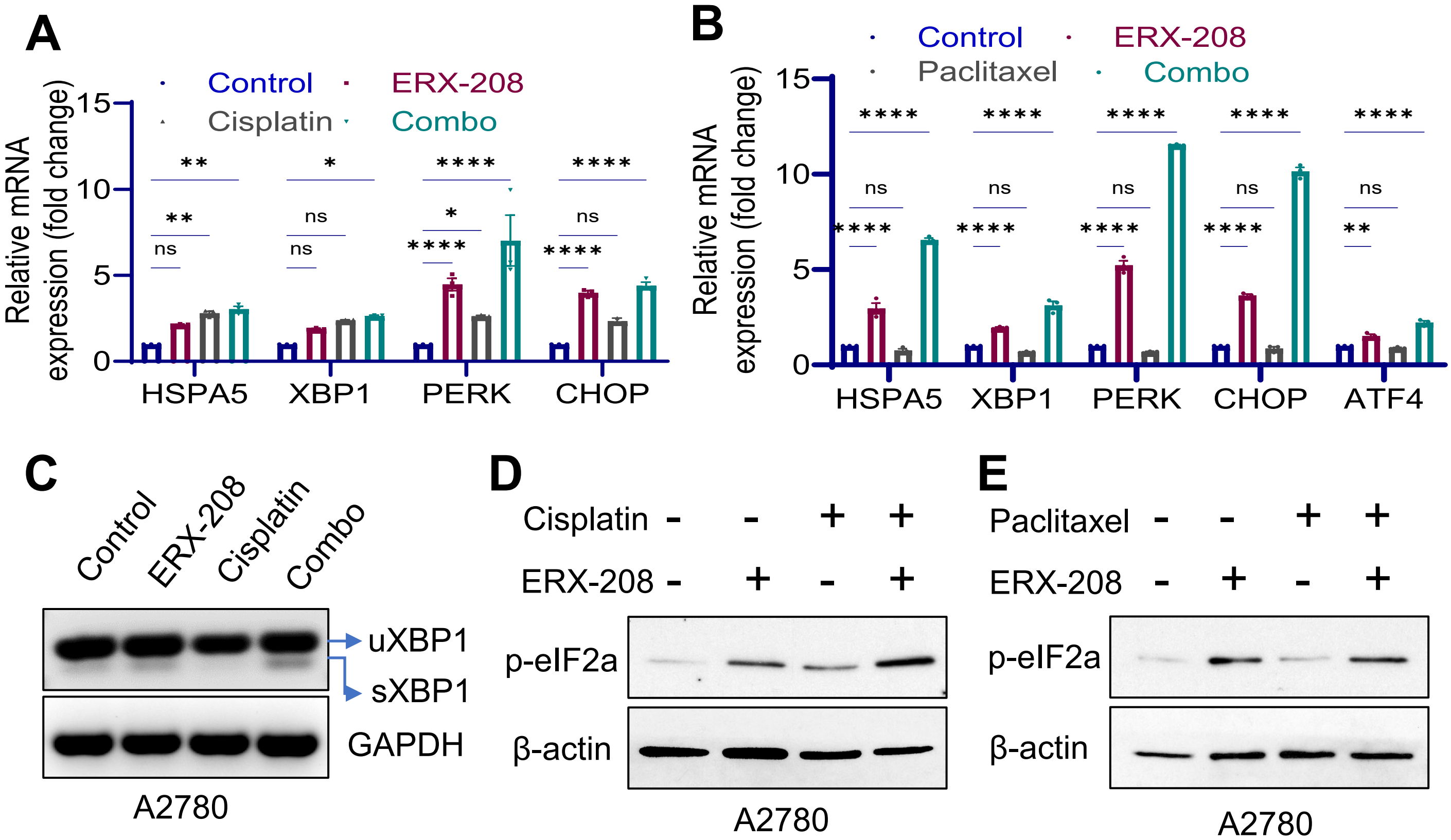

Supplementary Fig. 5. ERX-208 activates UPR signaling and enhances the anti-proliferative effects of chemotherapy in OCa cells. (A) Quantitative RT-PCR analysis of UPR-related genes (HSPA5/BiP, XBP1, PERK, and CHOP/DDIT3) in A2780 cells treated for 24 h with ERX-208 (200 nM), cisplatin (5 μM), or the combination. Combination treatment significantly increased expression of UPR target genes, particularly CHOP and XBP1. (B) mRNA expression of HSPA5, XBP1, PERK, CHOP, and ATF4 in A2780 cells treated with ERX-208 (200 nM), paclitaxel (5 nM), or the combination. ERX-208 enhanced paclitaxel-induced upregulation of multiple UPR markers, most prominently CHOP and XBP1. (C) Analysis of XBP1 splicing in A2780 cells treated with ERX-208 (200 nM), cisplatin (5 μM), or the combination. Increased levels of spliced XBP1 (sXBP1) indicate activation of the IRE1α-XBP1 arm of the UPR. (D) Western blot showing phosphorylation of eIF2α (p-eIF2α) in A2780 cells treated with cisplatin (5 μM), ERX-208 (200 nM), or the combination. β-actin serves as loading control. ERX-208 increased p-eIF2α alone and further enhanced cisplatin-induced phosphorylation. (E) Western blot analysis of p-eIF2α in A2780 cells treated with paclitaxel (5 nM), ERX-208 (200 nM), or both. ERX-208 augments paclitaxel-mediated activation of the PERK-eIF2α pathway. Data represent mean ± SEM; ns, not significant; *$p < 0.05$, **$p < 0.01$, ***$p < 0.001$, ****$p < 0.0001$.
