# Supplementary material for: ERX-208 Induces Endoplasmic Reticulum Stress and Suppresses DNA Damage Repair to Enhance Chemotherapy Sensitivity
Source: Cancer Res Commun. 2026 Oct 29;6(10):2541–54. doi: 10.1158/2767-9764.CRC-26-0421 (PMC13718282; doi:10.1158/2767-9764.CRC-26-0421)
Supplement: Supplementary file 6 [file crc-26-0421_supplementary_figure_s6_suppsf6.pdf]

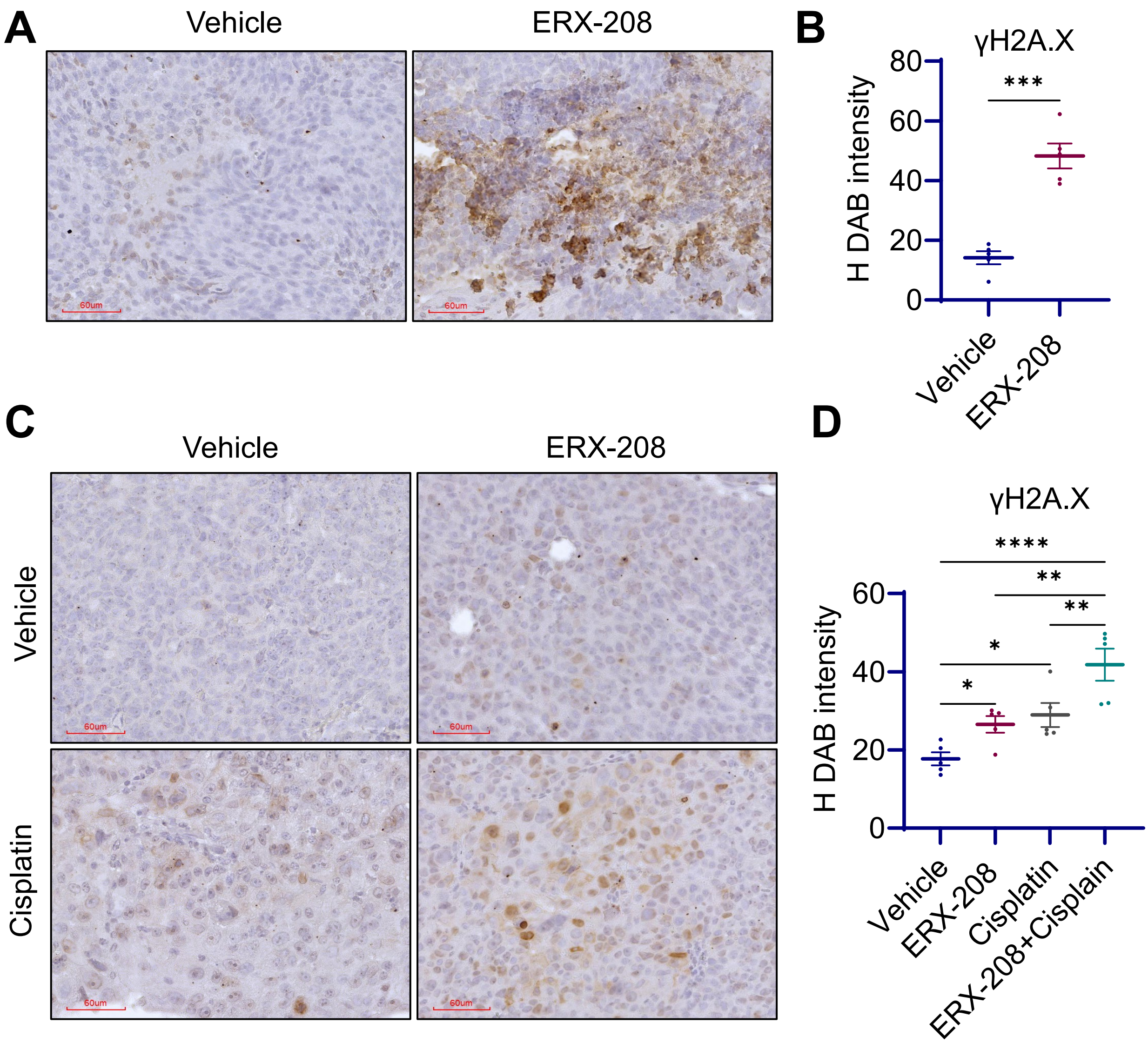

Supplementary Fig. 6. A-B, Increased expression of γH2A.X in ERX-208 treated group compared with vehicle control group in OCa-38 PDX model. (A) Representative immunohistochemical staining for tissue sections from Control group and ERX-208 group. (B) Quantification of γH2A.X positive staining intensity is shown. C-D, Treatment-induced DNA damage in OCa-67 PDX tumors assessed by γH2AX immunostaining. (C) Representative images demonstrate progressively increased γH2AX-positive nuclei in tumors from treated groups compared with control tumors. (D) The intensity of γH2AX staining was quantified using ImageJ software. Data are presented as mean ± SEM; *$p < 0.05$; **$p < 0.01$; ***$p < 0.001$ ****$p < 0.0001$. Scale bar = 60 μm.
